# The Areca Nut and Oral Submucosal Fibrosis: A Narrative Review

**DOI:** 10.3390/dj13080364

**Published:** 2025-08-12

**Authors:** Kimia Kazemi, Asmaa Fadl, Felipe F. Sperandio, Andrew Leask

**Affiliations:** College of Dentistry, University of Saskatchewan, 105 Wiggins Road, Saskatoon, SK S7N 5E4, Canada; kimia.kazemi@usask.ca (K.K.); asmaa.fadl@usask.ca (A.F.); felipe.sperandio@usask.ca (F.F.S.)

**Keywords:** Oral Submucous Fibrosis, betel nut, myofibroblasts, fibrosis, TGF-β, arecoline, CCN2

## Abstract

The areca nut (AN) is chewed by approximately 600 million people worldwide. Among AN chewers, ~5% develop oral submucosal fibrosis (OSF), a progressive fibrotic disorder of the oral cavity. OSF is characterized by subepithelial fibrosis and mucosal rigidity, leading to restricted mouth opening, difficulty in mastication, deglutition, and speech. These impairments severely compromise oral hygiene and routine dental care, diminishing patients’ quality of life. At least 4% of OSF patients develop oral cancer. The prevalence of OSF correlates with AN chewing, particularly when accompanied by other risk factors such as tobacco use. The International Agency for Research on Cancer has identified chronic chemical and mechanical irritation of the oral mucosa from AN chewing as a major cause of OSF. The active chemical ingredients of AN include alkaloids such as arecoline, flavonoids, and tannins. Of these, arecoline is considered the most potent fibrogenic agent. In vitro, arecoline induces cultured fibroblasts to differentiate into highly contractile α-smooth muscle actin (α-SMA)-expressing myofibroblasts, the effector cells of fibrosis, and to express profibrotic markers and mediators, including transforming growth factor-β 1 (TGF-β1) and cellular communication network factor 2 (CCN2), which is associated with malignant progression of OSF. In vivo, mice exposed to AN extract or arecoline show submucosal collagen accumulation and myofibroblast differentiation, concomitant with upregulated pro-fibrotic gene (TGF-β1, Col1A1, α-SMA) expression. Although myofibroblasts can be seen in OSF patient-derived samples, substantial disease heterogeneity exists, which has thus far hindered the generation of high-quality data necessary to gain insights into underlying mechanisms and disease progression. Consequently, treatment options for OSF are limited and primarily symptomatic. Collectively, evidence from human and animal studies establishes OSF as an AN-induced fibrotic disorder and underscores the urgent need for mechanism-focused research to identify reliable diagnostic markers and therapeutic targets to address its growing global burden.

## 1. Introduction

Oral submucosal fibrosis (OSF), caused by areca nut (AN, aka betel nut) chewing, is highly under-researched despite being a major health concern in South and Southeast Asia. Herein, we discuss what is known regarding the mechanisms underlying OSF, avoiding speculation that is unsupported by the literature, and suggest that high-quality, sufficiently powered molecular studies that consider disease heterogeneity are required.

In a rural village in Maharashtra, a 9-year-old girl who regularly helped her parents on their farm inherited the habit of chewing sweetened areca nut (AN) (supari) from them. Her family, who worked as cowherds, had long practiced areca nut chewing as part of their daily routine, and the girl began the habit alongside other children in the community. Several years later, she developed a burning sensation in her mouth and difficulties opening her jaws. She was subsequently diagnosed with Oral Submucous Fibrosis (OSF), a chronic, progressive, and potentially malignant disorder of the oral cavity associated with AN consumption [1]. In another corner of the world, a 38-year-old South Asian migrant laborer in the United Arab Emirates exposed to gutka, processed AN with added tobacco, since adolescence suffered from undiagnosed OSF for years before seeking care; his condition was hidden behind cultural normalization and policy neglect [2,3]. These individuals represent the overlooked human cost of OSF. Their stories are not isolated, but rather shared by millions living in vulnerable communities without access to timely diagnosis, education, or regulatory protection. This paper gives voice to them, not only through science but through advocacy.

AN has been categorized as a Group 1 carcinogen by both the World Health Organization and the International Agency for Research on Cancer, with strong evidence linking its use to OSF and cancers of the mouth, throat, and esophagus [4]. Despite its well-documented progression to cancer, with studies reporting that, among all oral premalignant conditions, it has one of the highest malignant transformation rates [5], OSF remains severely under-recognized in clinical guidelines, national cancer registries, and preventative dental programs [3,6]. OSF is predominantly observed in South Asia, where AN consumption is culturally ingrained, and is on the rise among South Asian migrant populations in the Middle East, where AN Products are sold with minimal regulation [2,3]. Therefore, in this paper we advocate for urgent attention, equitable healthcare, and global recognition of OSF as a preventable, socially driven disease rooted in cultural normalization, misinformation, and policy neglect.

Herein, we combine scientific findings with a call to action. We must ensure that OSF is recognized for what it is: a fibrosis-driven cancer precursor that mirrors the pathogenesis of systemic fibrotic diseases and deserves the same urgency, funding, and global policy response. Our advocacy is grounded in mechanistic and histopathological insights of OSF, including the central role of α-smooth muscle actin (SMA)-expressing myofibroblasts, transforming growth factor (TGF)-β, YAP/CCN2 signaling, and epithelial–mesenchymal transition (EMT), supported by current molecular evidence from RNA sequencing analysis and patient data developed through our team’s translational research efforts. Drawing on clinical parameters, as well as immunohistochemical and epidemiological evidence, we stress the need for integration of OSF into cancer screening, stronger regulation of AN products, and greater investment in research reflecting its pathological complexity. OSF is a matter of scientific priority and social justice—the time to act is now.

## 2. Epidemiology of OSF

It is estimated that 10 to 20% of the global population regularly chews betel quid, with approximately 600 million individuals worldwide chewing AN [7]. OSF is strongly associated with the habit of chewing AN, often in the form of betel quid, paan masala, or gutkha, which are widely chewed in South and Southeast Asian regions [8]. Epidemiological studies indicate that OSF is predominantly seen in countries such as India, Bangladesh, Sri Lanka, Pakistan, Taiwan, and southern China, with additional cases reported among Asian diaspora in Western countries, including the UK, South Africa, and North America, due to migration and changing cultural habits [9]. The prevalence of OSF varies widely depending on geographical location, cultural practices, and study methodologies, ranging from 0.1% to 30% in different populations [10].

OSF disproportionately affects males, with a male-to-female ratio ranging from 3:1 to 5:1 in most studies; female prevalence is rising in regions where AN consumption by women is increasing [11]. OSF typically manifests in adults aged 20–40 years but has been reported in children as young as 4, particularly in areas with early initiation of chewing habits [12]. This rising incidence among younger populations is a growing public health concern, driven by the accessibility and affordability of flavored AN products [13].

## 3. Cultural Roots and Public Blind Spots

Despite its classification as a Group 1 carcinogen by the WHO [4,14], AN is rarely framed as a tobacco-equivalent public health threat. In many South Asian communities, it is embedded in cultural and religious traditions, marketed as a digestive aid or mouth freshener, and consumed without stigma [7]. As a result, both patients and care providers often fail to associate AN consumption with disease risk. When individuals from these communities migrate, they bring the habit with them—but the healthcare systems in host countries often remain unaware [7]. Clinicians frequently ask, “Do you smoke or use tobacco products?”, and often, they do not realize that gutka, paan, or betel quid are relevant sources of exposure. This disconnect between cultural practices and clinical protocols leads to missed diagnoses, under-reporting, and a lack of early intervention. Moreover, OSF remains excluded from clinical trials, global burden assessments, and cancer prevention programs, reinforcing its status as a public health blind spot [7,12].

## 4. Clinical and Histopathological Parameters for Diagnosing OSF

### 4.1. Clinical Parameters

The diagnosis of OSF relies primarily on clinical evaluation, including patient history, visual inspection, and functional assessment [15]. Early symptoms often involve a burning sensation exacerbated by spicy foods, along with ulceration and pain [16]. Visual signs include mucosal blanching, palpable fibrous bands, mucosal stiffness, and loss of elasticity [16]. Restricted mouth opening (trismus), measured as reduced interincisal distance, is a key functional parameter, often accompanied by impaired tongue movement, chewing, swallowing, or speech [16]. A functional staging system based on interincisal mouth opening, such as that proposed by Chandramani More et al., categorizes patients into stages M1 (>35 mm), M2 (25–35 mm), M3 (15–25 mm), and M4 (<15 mm). This classification aids in evaluating disease progression and informing clinical management [17]. Other clinical features may include vesicles, ulcerations, petechiae, or pigmentation changes [15]. A detailed history of AN chewing (e.g., betel quid, paan masala, or gutkha) is critical, as it is the primary etiological factor. In advanced cases, involvement of the pharynx or esophagus may cause dysphagia [16].

### 4.2. Histopathological Parameters

While clinical features are often sufficient for diagnosis, histopathological examination provides confirmatory evidence, especially in ambiguous cases or to rule out malignancy. Early OSF shows epithelial changes such as atrophy, hyperplasia, or hyperkeratosis, with juxta-epithelial inflammation and edema in the lamina propria [15,16]. Disease progression is marked by dense collagen deposition, submucosal fibrosis, and hyalinization [15]. In advanced stages, fibrous bands replace normal connective tissue, causing reduced vascularity and muscle degeneration. Inflammatory infiltrates, mainly lymphocytes, are prominent early but diminish as fibrosis advances [15]. Moreover, dysplastic changes, such as nuclear atypia and increased mitosis, indicate malignant potential, with OSF transforming to OSCC in 1.5–15% of cases [18,19]. Collectively, histopathological grading generally correlates with clinical severity, informing prognosis and treatment; biopsy is recommended when malignancy is suspected or at early signs of clinical changes/transformation [15].

Despite established clinical and histopathological criteria, OSF often goes undiagnosed, under-reported, or misclassified, particularly in regions with limited healthcare infrastructure. In many high-risk communities, access to oral pathologists is scarce, and frontline providers may lack training in early detection [20]. Advocacy is needed to standardize and expand OSF screening through primary care and community health systems, especially in underserved populations. Early diagnosis is not just a clinical goal—it is a public health imperative. Importantly, OSF is insufficiently studied across both endemic and non-endemic regions.

### 4.3. Insights into the Molecular Basis of OSF: Myofibroblasts Differentiation and Malignant Transformation

The pathological effects of betel nut chewing on promoting oral cancer and periodontal disease were first reported in early 1950s [21,22,23], but studies on the molecular nature of OSF only began around 2011 and remain limited. OSF is similar to other fibrotic diseases where excessive deposition of scar tissue can lead to organ dysfunction; in contrast fibrotic conditions of the gingiva, such as gingival overgrowth (GO)/drug-induced gingival hyperplasia, are characterized not by scarring, but by a hyperproliferation [24]. Moreover, OSF has a higher malignant transformation rate than other potentially malignant oral lesions, such as certain types of leukoplakia, i.e., epithelial dysplsia, and actinic cheilitis [25,26].

**Figure 1:**
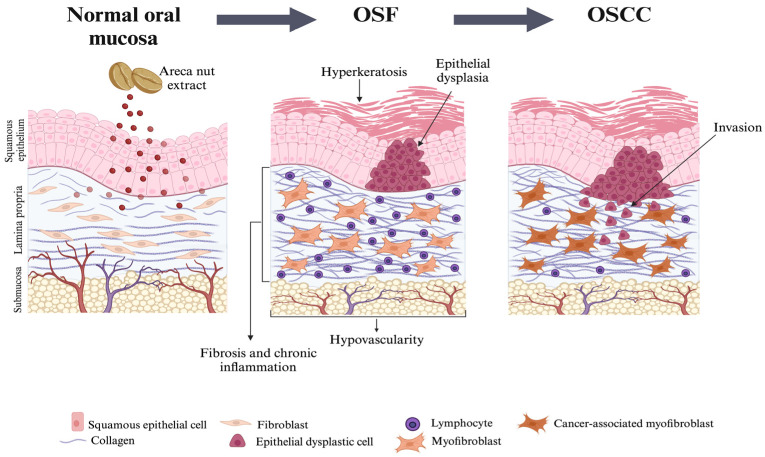
Histopathological features of normal oral mucosa, OSF, and OSCC. A schematic model is shown. Areca nut extract induces Oral Submucous Fibrosis (OSF), characterized by infiltration of connective tissue with chronic inflammatory cells, such as lymphocytes, excessive deposition of dense collagen, myofibroblast differentiation, and reduced vascularity, leading to fibrosis. Some cases also exhibit hyperkeratosis and epithelial dysplasia [8]. OSF has a tendency to progress to oral squamous cell carcinoma (OSCC), marked by the invasion of dysplastic epithelial cells into the connective tissue and the activation of cancer-associated myofibroblasts [8]. Figure made using BioRender.

The clinical presentation of OSF, resembling tissue scarring in other organs, suggests a pathogenic mechanism involving chronic inflammation, collagen deposition, and myofibroblast differentiation, with potential progression to OSCC (Figure 1) [27]. The α-SMA-expressing myofibroblasts likely drive the pathological ECM remodeling in OSF [28]. Conversely, gingival fibroblasts are relatively resistant to myofibroblast differentiation and are infrequently observed in GO [29,30,31,32,33]. The first study reporting myofibroblast presence in OSF compared tissues from 70 OSF patients at different fibrosis stages with 15 healthy controls, showing a significant increase in α-SMA-positive myofibroblasts in OSF tissues (*p* < 0.001), correlating with disease progression [34]. We also show herein an increased myofibroblastic population in OSF samples when compared to common reactive fibroblastic lesions of the oral cavity (see Supplementary Figure S1). Thus, OSF may be more appropriately classified among other myofibroblastic oral lesions, such as myofibroma, desmoplastic fibroma, and myofibroblastic sarcoma [35]. Based on this, we argue that the primary effector cell in OSF is the myofibroblast and, hence, that anti-fibrotic treatments targeting myofibroblast activity could offer a potential treatment approach.

**Figure 2:**
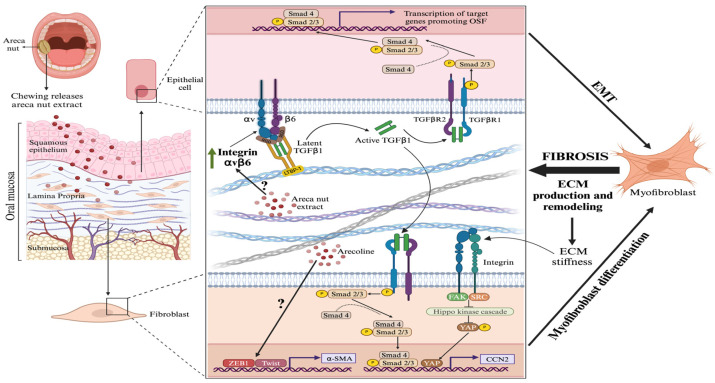
Proposed mechanisms of how areca nut extracts promote fibrosis. A data-derived schematic is shown. Areca nut extract, including the active ingredient arecoline, activate keratinocytes [through epithelial mesenchymal transition (EMT)] and fibroblasts [through the action of TGF-β and extracellular matrix (ECM) stiffness] to differentiate into myofibroblasts, the effector cell of fibrosis. For details, see text. Figure made using BioRender (https://www.biorender.com/, accessed on 18 July 2025). TGFβR = TGF-β receptor. FAK = focal adhesion kinase. YAP = yes-associated protein. SRC = proto-oncogene c-Src.

TGF-β is a major activator of myofibroblast differentiation in vitro and in vivo. In epithelial cells, AN extract and its derived alkaloid or polyphenol fractions increased TGF-β-induced transcripts and phosphorylated Smad2, a key downstream effector of TGF-β signaling (Figure 2) [36]. However, these effects did not result in human gingival fibroblasts [36]. Highlighting the importance of epithelial keratinocytes in driving OSF, increased expression of the integrin αvβ6—which, in epithelial cells, activates ECM-bound latent TGF-β—was observed in OSF patients (Figure 2) [37]. Immunohistochemistry confirmed α-SMA-positive myofibroblasts and active Smad signaling in both keratinocytes and myofibroblasts in tissues derived from OSF patients [37]. Similarly, arecoline, the major alkaloid of AN, upregulated integrin αvβ6 expression and active TGF-β in cultured keratinocytes, which caused differentiation of cocultured fibroblasts into myofibroblasts [37]. Notably, higher αvβ6 levels were seen in patients progressing to oral cancer [37]. These findings indicate that mucosal keratinocyte activation may drive OSF progression and malignant transformation.

Arecoline induces α-SMA expression in buccal mucosal fibroblasts through direct binding of the transcription factor ZEB1 to the α-SMA promoter (Figure 2) [38]. Another study similarly showed that arecoline induces α-SMA expression and promotes collagen gel contraction, a measure of myofibroblast activity, through the transcription factor Twist [39]. These changes are associated with cellular plasticity and activation of cancer-associated (myo)fibroblasts via EMT [40]. Additionally, Notch signaling, which promotes EMT, is elevated in OSF fibroblasts; siRNA-mediated Notch knockdown triggered apoptosis and suppressed EMT markers, effects rescued by exogenous TGF-β1 [41]. Although ZEB1, Twist, or α-SMA were not directly assessed after Notch inhibition, the findings support EMT pathways as drivers of myofibroblast activation in OSF [42]. The ECM in OSF is stiff, with increased collagen I and III expression and EMT induction in keratinocytes, as shown by atomic force microscopy [43]. OSF-derived ECM induced EMT in cultured keratinocytes, accompanied by activation of exogenous piezo-type mechanosensitive ion channel component 1 (Piezo1), and reduction by inhibition of the mechanosensitive transcriptional cofactor yes-associated protein (YAP) was also noted [43]. These findings support the idea that, as in other fibrotic diseases, ECM stiffness—maintained by a YAP-dependent mechanotransduction loop—is critical for fibrogenesis [44,45]. Several observations support this concept. First, siRNA targeting periostin, a TGF-β-regulated fibrotic mediator associated with mechanical stress, impaired α-SMA expression and collagen gel contraction in fibrotic buccal mucosal fibroblasts [46]. A later study confirmed the potential role of periostin in OSF, showing increased immunohistochemical staining in patient samples, particularly in advanced cases, where 78.57% (*n* = 14) exhibited periostin expression localized to fibrotic and hyalinized areas [47]. Second, in OSF patients, lower expression of the tumor and adhesive signaling repressor phosphatase and tensin homolog (PTEN) correlated with higher α-SMA expression, dysplasia, and OSCC [48], consistent with studies showing that fibroblast-specific PTEN loss promotes fibrosis and myofibroblast differentiation [49], suggesting PTEN loss contributes to OSF pathology

Data involving the pro-fibrotic matricellular protein CCN2 (formerly connective tissue growth factor) suggest a role for myofibroblasts in OSF pathogenesis. CCN2, a TGF-β and YAP target, when deleted from fibroblasts, rescues fibrosis caused by loss of PTEN expression [50,51,52]. Additionally, arecoline stimulates CCN2 production in buccal mucosal fibroblasts, a response suppressed by NF-κB, JNK, p38 MAPK inhibitors, and antioxidants [53,54] and mediated through the activation of latent TGF-β and mitochondrial-derived reactive oxygen species (ROS) (Figure 2) [54]. In mice, arecoline induces TGF-β and CCN2 expression, concomitant with induction of ROS and NLRP3 inflammasome-related factors [55]. In patient tissue, CCN2 expression correlates with OSF progression to OSCC, but it is absent in healthy tissues [56]. Collectively, these findings suggest that TGF-β/YAP/ROS-driven induction of CCN2 may contribute to OSF pathogenesis and malignant transformation, although this remains to be directly tested. The action of YAP may also involve bone morphogenic protein 4 (BMP4), as arecoline-induced epithelial mesenchymal transition of human oral keratinocytes is dependent on YAP-induced BMP4 [57]. Finally, as OSF is a fibrotic disease, it is characterized by the overproduction of type I collagen, linked with polymorphisms in lysyl oxidase, a critical collagen biosynthetic enzyme that is upregulated in OSF [58]. A summary of the molecular pathways implicated, and the type of supporting evidence supporting their role, in OSK is presented in Table 1.

## 5. Conclusions

The global burden of OSF remains incompletely characterized due to the lack of large-scale, well-designed epidemiological surveys. Existing studies often suffer from inconsistent diagnostic criteria and variable outcome measures, underscoring the need for standardized protocols to better understand the true prevalence and risk factors [59]. As AN consumption continues to spread globally, particularly among immigrant communities, public health efforts must focus on education, early detection, and cessation programs to mitigate the rising incidence of OSF and its associated malignancies [60].

Despite advances in understanding OSF’s molecular drivers, it remains under-recognized in clinical and public health contexts. OSF is rarely screened for, absent from cancer registries, and overlooked in global oral health policies. Cultural practices, healthcare gaps, and policy neglect contribute to its continued underdiagnosis. Our integrated analysis of tissue-level immunohistochemistry and transcriptomic profiling provides compelling evidence that OSF is an active fibrotic disease, characterized by myofibroblast activation, ECM remodeling, and upregulation of TGF-β/YAP/CCN2 signaling pathways. Although based on a small cohort, our findings reveal patterns that align with fibrotic remodeling processes observed in systemic diseases, demonstrating that meaningful molecular insights can still emerge from limited but well-characterized samples. Critically, our results also highlight a broader challenge in OSF research: the lack of harmonized, histologically validated patient datasets. That said, the available evidence suggests that, from a translational perspective, anti-fibrotic drugs targeting canonical and non-canonical TGF-β signaling, including YAP-mediated mechanotranduction, and antioxidants (that target ROS generation) warrant further exploration.

Existing public resources, including GEO, often suffer from inconsistent diagnostic annotation, variable staging, and insufficient clinical metadata, making it difficult to compare findings or build predictive models. To properly interpret data derived from OSF patients, clinical parameters such as age, sex, disease duration, histological stage, and lesion location must be systematically recorded at the time of sample collection. Moreover, to properly capture disease heterogeneity, large numbers of patients need to be analyzed. As an example of this problem, we recently examined publicly available databases and found that, for example, GSE274203 was poorly annotated and lacked description of patient parameters, contributing to widely divergent gene expression profiles between the two OSF patients analyzed and an inability to draw accurate conclusions with this underpowered dataset (See entire Supplemental file, including Supplemental Table S1 and Supplemental Figure S1. Future efforts should prioritize the development of larger, stage-matched, and diagnostically consistent OSF cohorts with integrated clinical, histopathological, and transcriptomic profiling that are sufficiently powered to draw conclusions. Such datasets are essential not only for understanding disease progression but also for identifying actionable targets for early intervention.

Finally, we emphasize that OSF should be recognized globally as a neglected public health issue. Addressing AN consumption through culturally sensitive education and regulation, integrating OSF screening into oral cancer prevention, and training primary care providers to detect early signs are critical. Dedicated research, surveillance, and policy reform are urgently needed to close existing gaps and improve outcomes for affected communities.

## Figures and Tables

**Table 1:** Summary of molecular pathways and mediators that have been reported to be implicated in the pathogenesis of OSF, along with the type of the available supporting evidence. Evidence includes in vitro studies, in vivo models, patient tissue (IHC), and gene expression profiling studies.

Pathways/Molecular Mediators	Role in Pathogenesis	Available Supporting Evidence				References
		in vitro	in vivo	IHC on Patient Sampels	Gene Expression Profiling	
TGF-β1/Smad2 signaling	Major activator of myofibroblast differentiation	ü		ü	ü	[36,37,54]
TGF-β1/Smad3 signaling		ü	ü			[55]
TGF-β1/Smad4 signaling				ü		[37]
Integrin αvβ6	Activation of TGF-β/Smad pathway and myofibroblast differentiation	ü		ü		[37]
α-SMA	Highly expressed by myofibroblasts, which drive pathological ECM remodeling, excessive collagen production, and tissue contraction	ü	ü	ü	ü	[34,37,38,48,55]
CCN2	Pro-fibrotic matricellular protein that promotes ECM production and cooperates with TGF-β signaling to sustain fibrotic responses	ü	ü	ü	ü	[53,54,55,56]
COL1A1	Drives the production of type I collagen, leading to excessive ECM deposition, a hallmark of fibrosis	ü	ü	ü	ü	[47,55,58]
YAP1/BMP4 signaling	Promoting EMT, and YAP is mechanosensitive transcription cofactor that promotes pro-fibrotic gene expression like CCN2	ü				[57]
PTEN	Less PTEN expression has been correlated with higher a-SMA expression at tissue staining level			ü		[48]
Periostin	Pro-fibrotic matricellular protein and cell adhesion molecule, promoting collagen fibrillogenesis and tissue stiffness	ü		ü	ü	[46,47]

## Supplementary Materials

The following supporting information can be downloaded at: https://www.mdpi.com/article/10.3390/dj13080364/s1, Figure S1 Heatmap visualization of differentially expressed genes associated with ECM remodeling and cell signaling pathways in OSF versus normal oral mucosa (NOM) based on GSE274203 RNA-seq data; Table S1: Pathway Enrichment Analysis of Upregulated Genes in OSF. In file entitled “Lessons learned while attempting to investigate the molecular basis of OSF using a publically available gene expression dataset.

## Abbreviations

The following abbreviations are used in this manuscript:

AN	Areca nut
OSF	Oral submucosal fibrosis
SMA	Smooth muscle actin
TGF	Transforming growth factor
YAP	Yes-associated protein
