# Supplementary material for: The Areca Nut and Oral Submucosal Fibrosis: A Narrative Review
Source: Dent J (Basel). 2025 Aug 12;13(8):364. doi: 10.3390/dj13080364 (PMC12385254; doi:10.3390/dj13080364)
Supplement: Supplementary file 1 [file dentistry-13-00364-s001.zip › dentistry-3735577-supplementary.pdf]

Supplemental File

Lessons learned while attempting to investigate the molecular basis of OSF using a publicly available gene expression dataset.

OSF is a neglected precancerous condition and a growing global public health concern, yet there remains a striking lack of in vitro, in vivo, and transcriptomic studies across its clinical stages and key biological variables, including patient age, sex, and duration or frequency of areca nut consumption .

In this supplementary file, we highlight GSE274203, a publicly available gene expression dataset, as a clear example of the field's current limitations—an underpowered dataset with only two OSF samples exhibiting high inter-patient variability. Our functional cluster analysis using this dataset underscores the difficulty in drawing reliable conclusions from such sparse and heterogeneous data.

This reinforces the central aim of our paper: to call for urgent, mechanism-focused research using well-characterized in vivo models and OSF patient samples, stratified by disease stage and key biological factors such as age, sex, and duration of areca nut use. Comprehensive gene expression profiling is essential to advance our understanding of OSF pathogenesis and to guide the development of effective diagnostic and therapeutic strategies. Furthermore, global coordination in research efforts and regulatory action is critical, given that areca nut is classified as a Group 1 carcinogen by the WHO yet remains largely unregulated in many regions.

1. Transcriptomic Profiling Methodology

1.1 OSF Data Preparation

To investigate differentially expressed genes (DEGs) in OSF, we analyzed a gene expression profile, GSE274203, obtained from the Gene Expression Omnibus (GEO) database. It includes a total of 2 OSF samples, 2 OSCC samples, and 2 healthy control samples.

1.2 Identification of DEGs

After downloading the GEO datasets, raw expression data were processed and normalized. Differential gene expression analysis was performed using the DESeq2 package in R. Genes with an adjusted p-value < 0.05 and log2 fold change > 1.5 were considered significantly differentially expressed.

1.3 Functional Cluster Analysis

To explore the biological significance of the identified DEGs, we conducted Gene Ontology (GO) and Kyoto Encyclopedia of Genes and Genomes (KEGG) pathway enrichment analyses using the The Database for Annotation, Visualization, and Integrated Discovery (DAVID) online tool. GO

analysis classified the DEGs according to their associated biological processes (BP), molecular functions (MF), and cellular components (CC). KEGG analysis was employed to identify the biological pathways significantly enriched among the DEGs.

2. Results

To further investigate the molecular mechanisms underlying the myofibroblast activity observed in OSF tissues, we conducted transcriptomic analysis using the publicly available dataset GSE274203. This dataset includes samples from two OSF patients and two healthy individuals. Our aim was to determine whether the fibrotic phenotype observed clinically and histologically is reflected in the gene expression landscape.

When the expression data from the two OSF patients and two healthy individuals were combined and subjected to differential gene expression analysis identified 20,229 genes, of which 4,831 were upregulated in OSF samples compared to healthy mucosa. Among these, 705 genes met statistical significance ($p \leq 0.05$) and were subjected to functional cluster analysis using DAVID (Table I). The most significantly enriched pathway was the ECM cluster (enrichment score: 19.96), which included genes involved in collagen synthesis, matrix crosslinking, and structural remodeling—consistent with the fibrotic architecture seen histologically. Additional clusters included Focal Adhesion (enrichment score: 8.38), Growth Factor Signaling (enrichment score: 6.56), Basement Membrane Organization (enrichment score: 6.30), and Collagen Trimer Formation (enrichment score: 5.28). Other notable clusters such as Metalloendopeptidase Activity, Fibronectin Type-III, and Integrin-Mediated Signaling were also elevated, reflecting dynamic ECM remodeling and sustained myofibroblast activation. However, when heat maps were constructed of the underlying data, we found that the samples from the two OSF patients were the most dissimilar (Figure 1). Samples from one patient were responsible for the observed results, reflecting disease heterogeneity.

3. Conclusions

The dataset available from this study (GSE274203) is significantly underpowered by its small sample size (N = 2 OSF, N = 2 healthy), which limit the generalizability of the findings and the ability to draw a solid conclusion. Notably, inter-patient heterogeneity was also evident in the transcriptomic profiles where only one OSF patient sample appeared to drive a significantly larger

portion of the observed effect. In addition, visual inspection of heatmaps and sample clustering clearly show that one OSF patient sample contributed disproportionately to the observed differential expression and pathway enrichment. As such, it is possible that some findings reflect sample-specific variation rather than generalizable molecular features of OSF. While DESeq2 and DAVID are widely used tools, their application to datasets with very small sample sizes carries inherent limitations. Batch effects, sample-specific bias, and inflated fold changes may occur.

With such a small sample size (N = 2), any differences between the two patients, including such as age, disease severity, or other personal factors, can strongly influence the data and makes it hard to distinguish true disease-related gene expression changes from random individual variation, potentially skewing or distorting the transcriptomic findings. To support this contention, a histological study of tissue derived from 15 healthy controls and histologically-categorized 35 "early" OSF and 35 "advanced" OSF patients found significantly increased myofibroblast numbers (as detected using an α-SMA antibody) below the basal epithelial layer in 20/35 advanced cases showing high α-SMA staining, whereas minimal myofibroblast numbers were present in the remaining 15 "advanced" OSF patients showing moderate to low expression and in the 35 "early" OSF patients [Angadi PV, Kale AD, Hallikerimath S. Evaluation of myofibroblasts in oral submucous fibrosis: correlation with disease severity. J Oral Pathol Med. 2011 Mar;40(3):208-13].

Considering this substantial disease heterogeneity, to properly interpret data derived from OSF patients, clinical parameters such as age, sex, disease duration, and presentation need to be recorded at the time samples are collected, along with sufficient samples need to be analyzed to ensure robust conclusions. Moreover, future studies with larger, stage-stratified OSF cohorts and external validation—such as qPCR, IHC quantification, or independent transcriptomic datasets along with in vivo and large cohorts are essential to confirm these findings.

Table S1. Pathway Enrichment Analysis of Upregulated Genes in OSF. Pathway enrichment analysis of upregulated genes in OSF highlights key mechanisms driving fibrosis progression. The extracellular matrix (ECM) pathway is the most enriched, featuring FN1, TNC, COL4A2, and MMP13, indicating ECM remodeling and stiffness. Focal adhesion and integrin-mediated signaling pathways (ITGB1, ITGAV, FN1, PDGFB) reinforce fibroblast adhesion and cellular signaling. Growth factor signaling (IL11, PDGFB) promotes fibroblast proliferation and differentiation, while collagen trimerization (COL4A2, COL4A1) and basement membrane remodeling suggest increased ECM deposition. Metalloendopeptidase activity (MMP13) highlights ECM turnover, and MAPK signaling (PDGFB, IL1A) links growth factors to fibroblast activation. The upregulation of these pathways suggests a persistent pro-fibrotic and ECM-driven microenvironment in OSF.

Clusters	Enrichment score	Genes
**Extracellular matrix**	19.96	SERPINA1, COL16A1, TNC, LAMC2, DMP1, HAPLN1, LOXL2, PODNL1, ADAMTS6, TGM2, POSTN, COL27A1, EGFL6, MMP1, P3H1, MMP9, MMP10, MMP11, MMP13, COL4A2, COL4A1, COL4A5, COL8A1, MMP19, ANGPTL4, FBN2, COL17A1, LAMA1, COL11A1, LAMA3, ADAMTS12, THBS1, LTBP1, ACAN, WNT6, ADAMTS14, FLRT2, CPA6, CTHRC1, WNT10A, LAMB3, WNT7B, FN1, BMP1, COL5A1, COL7A1, COL5A2, MFAP2, TGFBI, AGRN
**Focal adhesion**	8.38	ITGB1, SHC1, ITGB4, LAMA1, LAMA3, RASGRF1, PDGFB, TNC, PDGFA, LAMC2, THBS2, THBS1, EGFR, CCND2, IBSP, SPP1, RAC2, FLNA, ITGAV, ITGB6, LAMB3, ITGA3, ACTN1, FN1, VEGFC, COL4A2, COL4A1, COL4A5, ITGA6, ITGA5, MET, BIRC2, BIRC3
**Growth factor**	6.56	IL11, CSF3, CSF2, PDGFB, BMP8A, VEGFC, PDGFA, BMP8B, TGFA, CXCL1, NRG1, INHBA, NGF, FGF1, BMP1, ARTN, CCN6, TIMP1, AMH
**Basement membrane**	6.29	COL17A1, EGFL6, COL4A2, LAMB3, COL4A1, LAMA1, COL7A1, LAMA3, COL4A5, COL8A1, LAMC2, LOXL2
**Collagen trimer**	5.28	COL17A1, COL27A1, COL16A1, COL13A1, MMP1, COL11A1, PLOD3, COL4A2, COL5A1, COL4A1, RPS18, COL7A1, C1QTNF6, COL5A2, SERPINH1, COL4A5, COL8A1, TIMP1, CTHRC1
**Metalloendopeptidase activity**	3.56	ADAMDEC1, MMP1, ADAMTS12, MMP9, MMP10, ADAM19, MMP11, ADAMTS14, MMP13, BMP1, ADAM12, MMP19, TLL2, ADAM8, TLL1, ADAMTS6
**Fibronectin type-III 3**	3.20	PTPRU, DSCAM, ITGB4, COL7A1, IL31RA, TNC, FN1, PTPRK, PTPRH, IL13RA2, SDK2, IL12RB2
**Integrin-mediated signaling pathway**	2.81	ITGB1, ADAMDEC1, COL16A1, ITGB4, ITGA3, FN1, ISG15, DIAPH3, ADAM12, ADAM8, ITGAV, ITGA6, ITGB6, ITGA5
**MAPK signaling pathway**	2.14	NGFR, ANGPT2, RASGRF1, PDGFB, VEGFC, PDGFA, RRAS2, TGFA, NGF, FGF1, DUSP9, EGFR, CDC25B, IL1A, RRAS, ARTN, IL1B, RAC2, FLNA, MET

Extracellular Matrix Gene Cluster (OSF vs healthy, GSE274203)

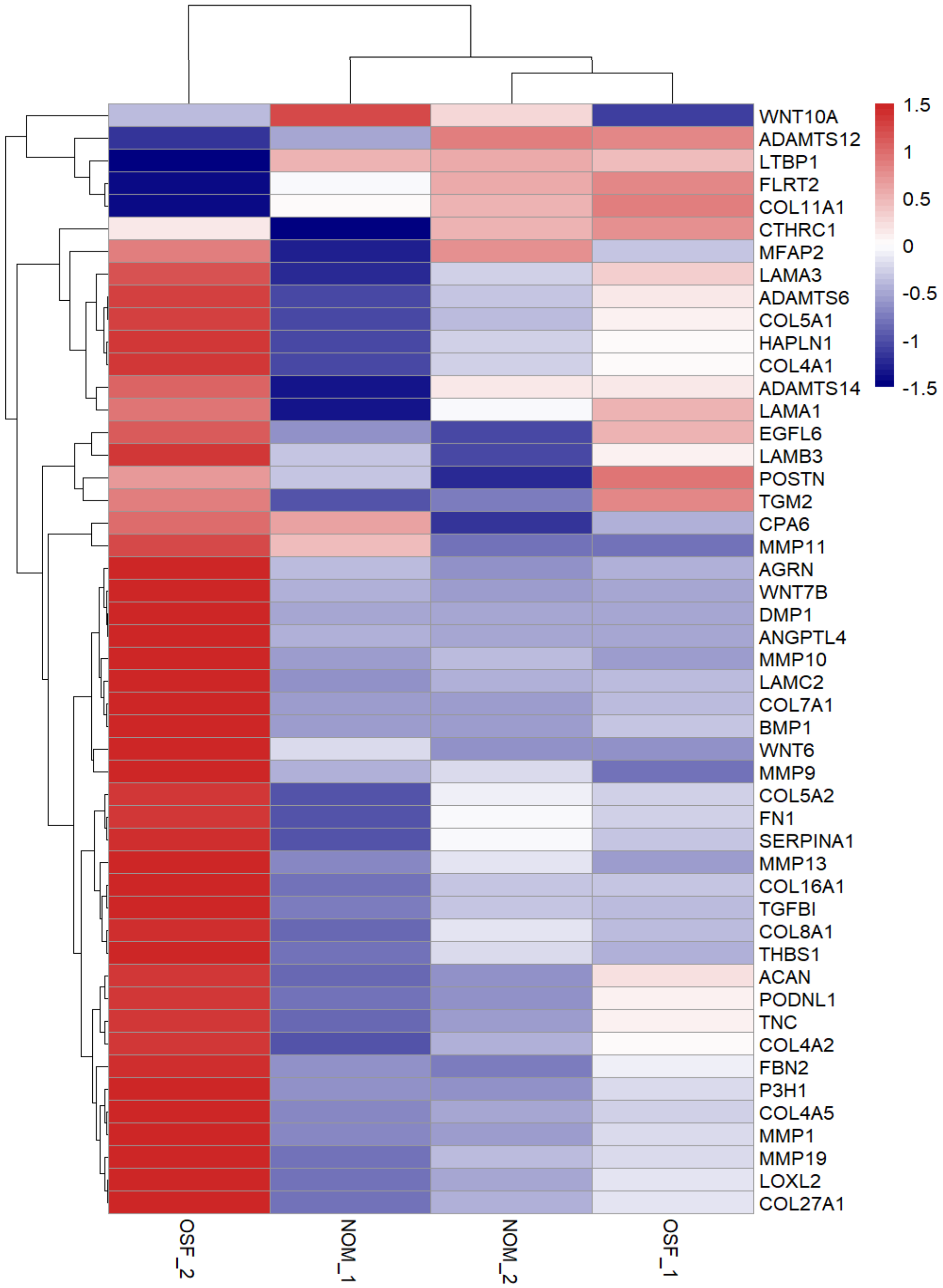

Focal Adhesion Gene Cluster (OSF vs healthy, GSE274203)

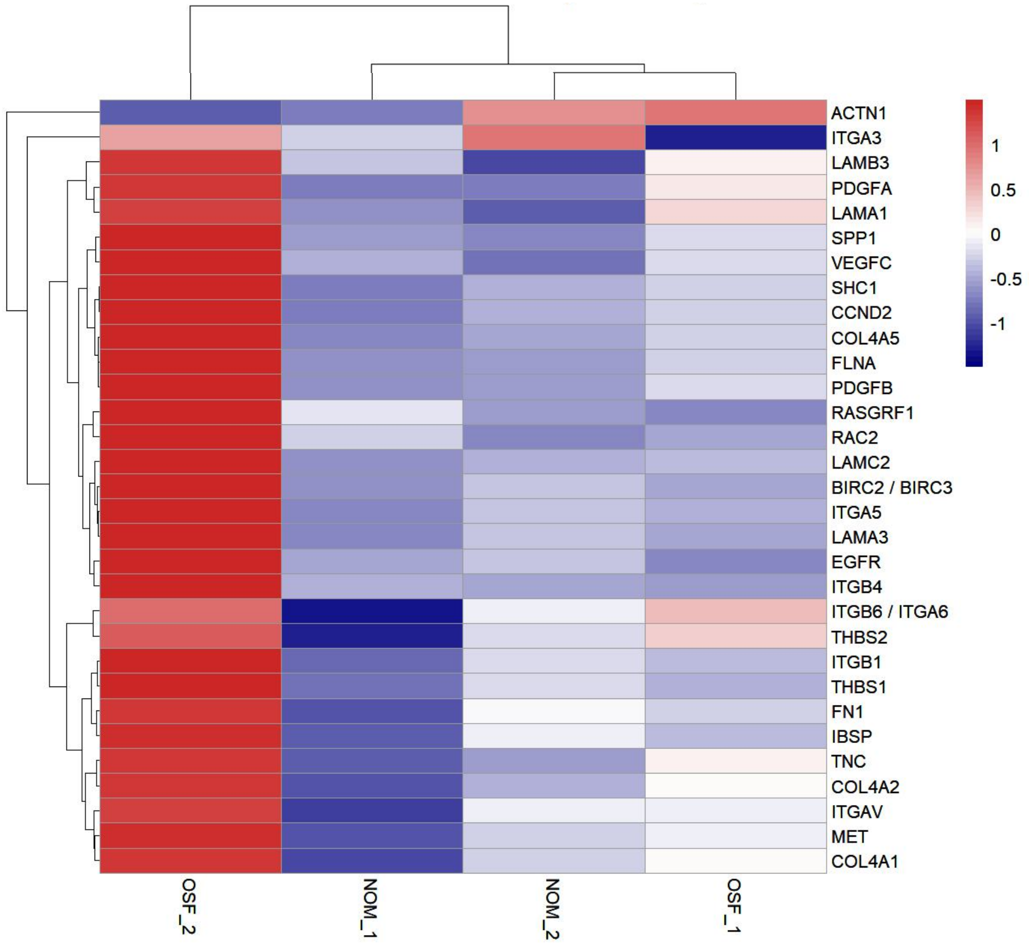

Growth Factor Gene Cluster (OSF vs healthy, GSE274203)

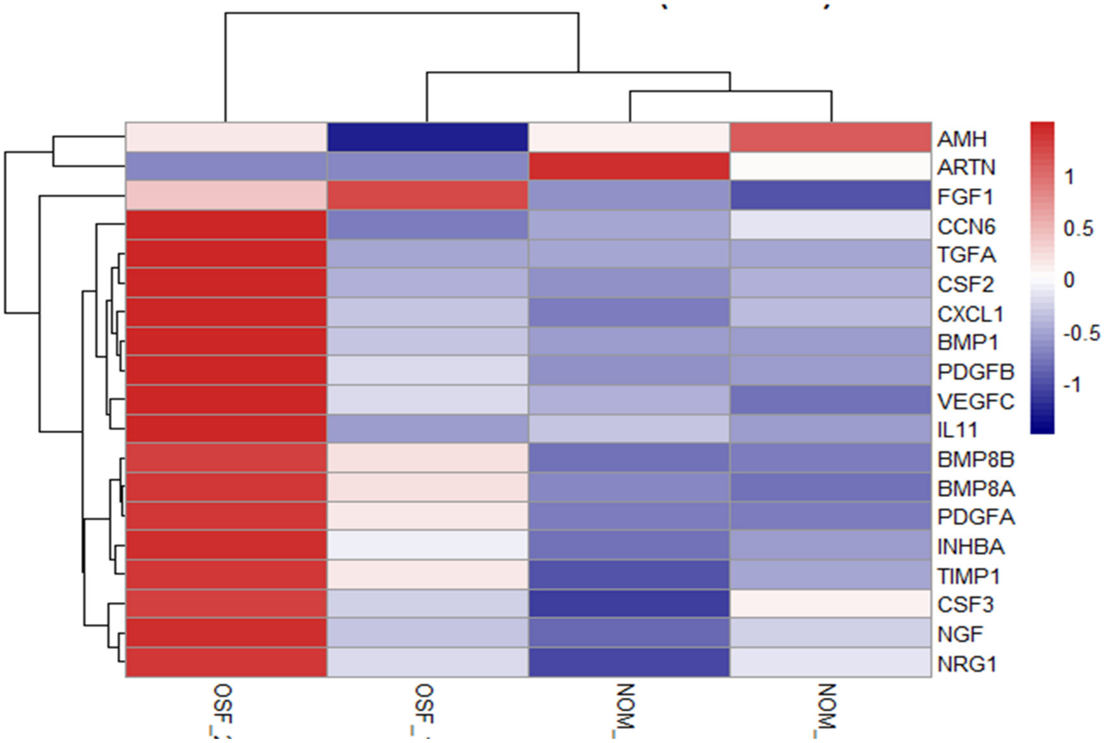

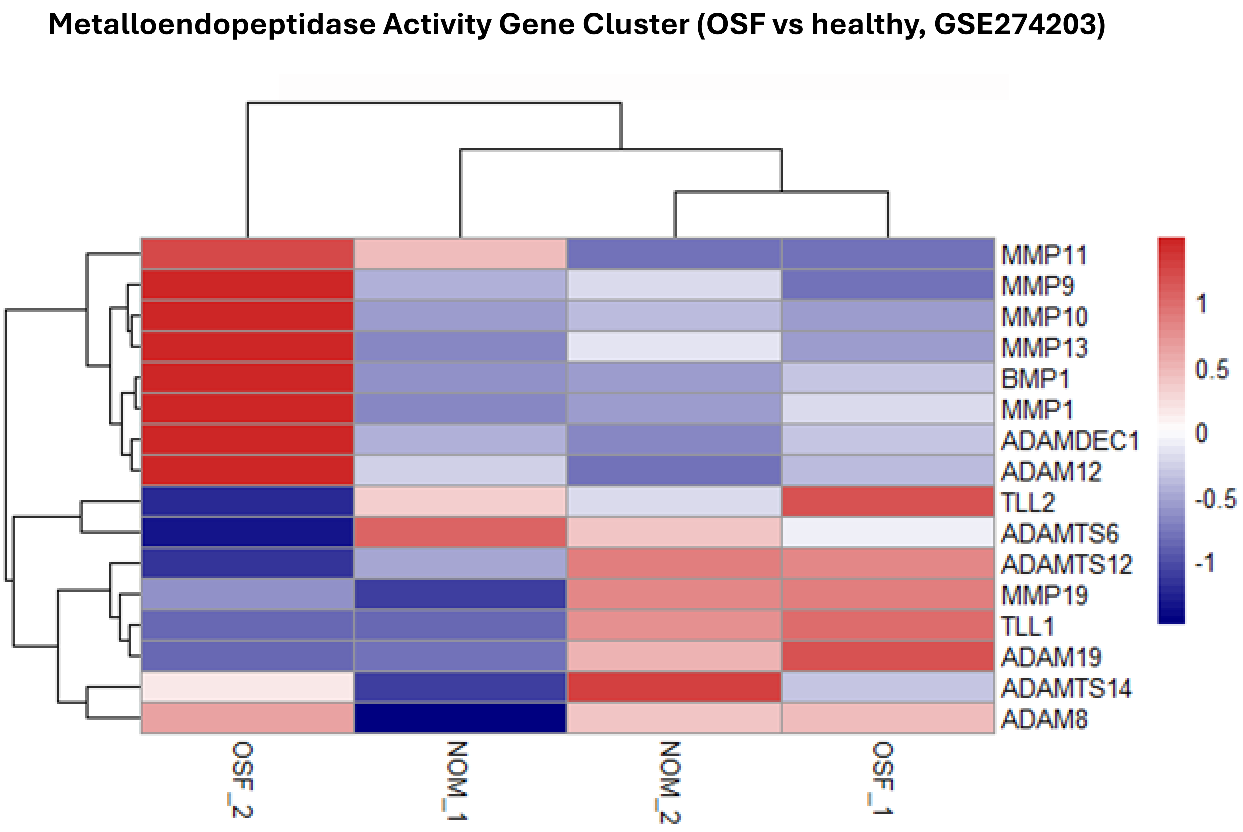

Figure S1. Heatmap visualization of differentially expressed genes associated with ECM remodeling and cell signaling pathways in OSF versus normal oral mucosa (NOM) based on GSE274203 RNA-seq data. (A) ECM structural components and remodeling-related genes, including collagens, laminins, and LOXL2, show distinct expression patterns between OSF and NOM tissues. (B) Genes associated with focal adhesion and cytoskeletal regulation demonstrate altered expression in OSF compared to NOM. (C) Growth factor-related genes show upregulation in OSF, including AMH, ARTN, and FGF1. (D) Metalloendopeptidase family genes, including several MMPs and ADAMTS members, are differentially regulated, highlighting ECM degradation activity. Expression values are row-scaled log2-transformed counts. Hierarchical clustering was performed across both genes and samples. Red indicates upregulation and blue indicates downregulation. Note disease heterogeneity in that, whereas healthy controls (NOM) showed similar expression patterns, the OSF samples showed high divergence. Altered gene expression observed obtained in one patient (OSF2) were sufficient to cause the results observed in Table 1.
